# Are beta blockers effective in preventing stroke-associated infections? - a systematic review and meta-analysis

**DOI:** 10.18632/aging.204086

**Published:** 2022-05-18

**Authors:** Li Yang, Xiang Wenping, Zhang Jinfeng, Pang Jiangxia, Wang Jingbo, Wang Baojun

**Affiliations:** 1 Department of Neurology, Baotou Center Hospital, Inner Mongolia, Baotou, China; 2 School of Medicine, Inner Mongolia Medical University, Inner Mongolia, Hohhot, China

**Keywords:** adrenergic beta-antagonists, stroke, infections, pneumonia, urinary tract infections

## Abstract

Background: Excessive sympathoexcitation could lead to stroke associated infection. Inhibiting sympathetic excitation may reduce the infection risk after stroke. Thus, the present study aimed to determine the protective effect of beta blockers on stroke associated infection through systematic review and meta-analysis.

Methods: A systematic search of multiple databases were performed up to February 2022. The included studies required beta blockers therapy in stroke patients and assessed the incidence of stroke-associated infections. Outcomes of interest included infections, pneumonia, urinary tract infection and sepsis. Random-effects model was used for analysis. Heterogeneity was evaluated using I2 statistics and publication bias was evaluated by the funnel plot.

Result: A total of 83 potentially relevant publications was identified in the initial search. Six studies met the inclusion criteria for meta-analysis. The risk of bias in the included articles satisfies the quality requirement of meta-analysis. No significant associations between beta blockers therapy and the prevention of stroke associated infection, stroke associated pneumonia and septicemia were found, However, subgroup analyses revealed an association between beta blockers treatment and the increased risk of post-stroke urinary tract infection or stroke associated pneumonia in some stroke patients (OR = 1.69 [1.33, 2.14], *P* < 0.0001; OR = 1.85 [1.51, 2.26], *P* < 0.0001).

Conclusion: Due to the lack of robust evidence, this meta-analysis may not support the preventive effect of beta blockers on stroke associated infection. But beta blockers treatment may be associated with development of post-stroke urinary tract infection and stroke associated pneumonia in some stroke patients.

## INTRODUCTION

Stroke has become the third leading cause of death and the main cause of permanent disability worldwide [1, 2]. Infection is one of the most frequent complications of stroke [3]. Stroke-associated infections (SAI) are defined as any infections diagnosed during the hospitalization period and the rates of SAI have been reported to up to 30% [4]. Stroke-associated pneumonia (SAP) and post-stroke urinary tract infection (UTI) are the two most common types of SAI [5–7]. Infections after stroke eventually increase the 30-day mortality rate and extend the hospital stay, and patients bear higher medical costs [5, 8].

Although the mechanism of SAI is unclear, it is believed that SAI are related to the excessive sympathoexcitation. First, stroke can disrupt the blood-brain barrier and lead to antigenic exposure of the central nervous system (CNS) [9], then, the immune system attacks exposed antigens leading to nerve cell damage [10]. Thus, stroke induced immunodepression syndrome (SIDS) can mitigate the immune response and protect nerve cells, but SIDS could increase the risk of SAI [6, 11]. Furthermore, recent researches have proven that sympathetic system activity is associated with SIDS [12]. For instance, sympathoexcitation could increase adrenaline secretion after stroke, which could decrease the number of T lymphocyte cells and disrupt the balance of T lymphocyte subgroups [13]. However, due to the absence of the β2-adrenergic receptor (2-AR) on Th-2 cells, catecholamine could hinder naive T lymphocyte differentiation into Th-1 cells but have no impact on Th-2 cells [14]. In addition, excessive sympathoexcitation could also break the immune balance by suppressing immune responses in the bone marrow, thymus, and spleen [6]. Thus, these changes in immune system could ultimately increase the risk of infection [12].

Theoretically, inhibiting sympathoexcitation may reduce the risk of infection by mitigating post-stroke immunosuppression. This hypothesis was experimentally confirmed in both animal experiments and clinical trials. In the mouse model of stroke, beta blockers could prevent immunological dysfunction and mitigate the risk of gram-negative and gram-positive bacterial infection [13, 15]. However, in the clinical trials, the effectiveness of beta blockers for preventing stroke associated infection is inconclusive. While some studies have showed that using beta blockers after the stroke could reduce the risk of SAI [9, 16], others have demonstrated the opposite or negative effects [16–18].

Moreover, many stroke patients had coexisting cardiac disease or hypertension, therefore, some patients may still use beta blockers to treat the cardiac disease after stroke [19, 20]. Therefore, it is still necessary to investigate the effect of beta blockers on SAI, regardless beta blocker treatment will have a beneficial or adverse effect.

Thus, exploring whether beta blockers are effective in preventing SAI can provide more valuable suggestions for clinical medication. To date there are no meta-analysis on the effect of beta blockers on stroke associated infections. Therefore, this study set out to determine the protective effect of beta blockers on stroke associated infection through systematic review and meta-analysis.

## METHODS

This meta-analysis followed the Cochrane and Preferred Reporting Items for Systematic Reviews and Meta Analyses (PRISMA) statement (Supplementary Table 1) [21]. This review was not registered.

### Search strategy

Two researchers (LY, ZJ) independently searched the databases including Web of Knowledge, PubMed, EMBASE, Medline and Cochrane Central Register of Controlled Trials to identify the English records. In order to obtain more comprehensive records and reduce the bias caused by ethnic differences. Two researchers searched database in other languages, such as China National Knowledge Infrastructure, WanFang data and Chinese biomedical database. In addition, reference lists of relevant review articles, clinical trial registers and eligible primary studies were manually scanned to retrieve potentially relevant studies. Databases were searched from inception up to February 2022.

The Mesh term for each search term was searched in combination with all the English synonyms associated with the respective Mesh term. The search strategies were constituted by three following groups of text or MeSH terms: (1) “stroke”, “ischemic stroke”, “brain infarction” or “cerebral hemorrhage”; (2) “infection”, “fever”, “pneumonia”, “urinary tract infection”, “sepsis” or “bacteremia”; and (3) “adrenergic beta-antagonists”, “beta blocker”, “adrenergic beta blocker”. Detailed search strategies are supplied in the Supplementary Table 2.

### Selection criteria

The retrieved studies were judged as eligible if they meet the following PICOS inclusion criteria: 1. The included patients who were diagnosed as stroke, regardless of the type of stroke, the diseased brain area, and the race, but the sample need to exclude the post-traumatic intracranial hemorrhages. 2. Patients received beta blocker therapy after stroke, if beta blockers were used prior to the stroke and without specified information about the beta blockers treatment after stroke, the patients are also considered as receive beta blockers therapy after stroke. 3. Patients developed infections such as pneumonia, UTI, sepsis or other infections after stroke. 4. Any prospective or retrospective studies.

**Figure 1:**
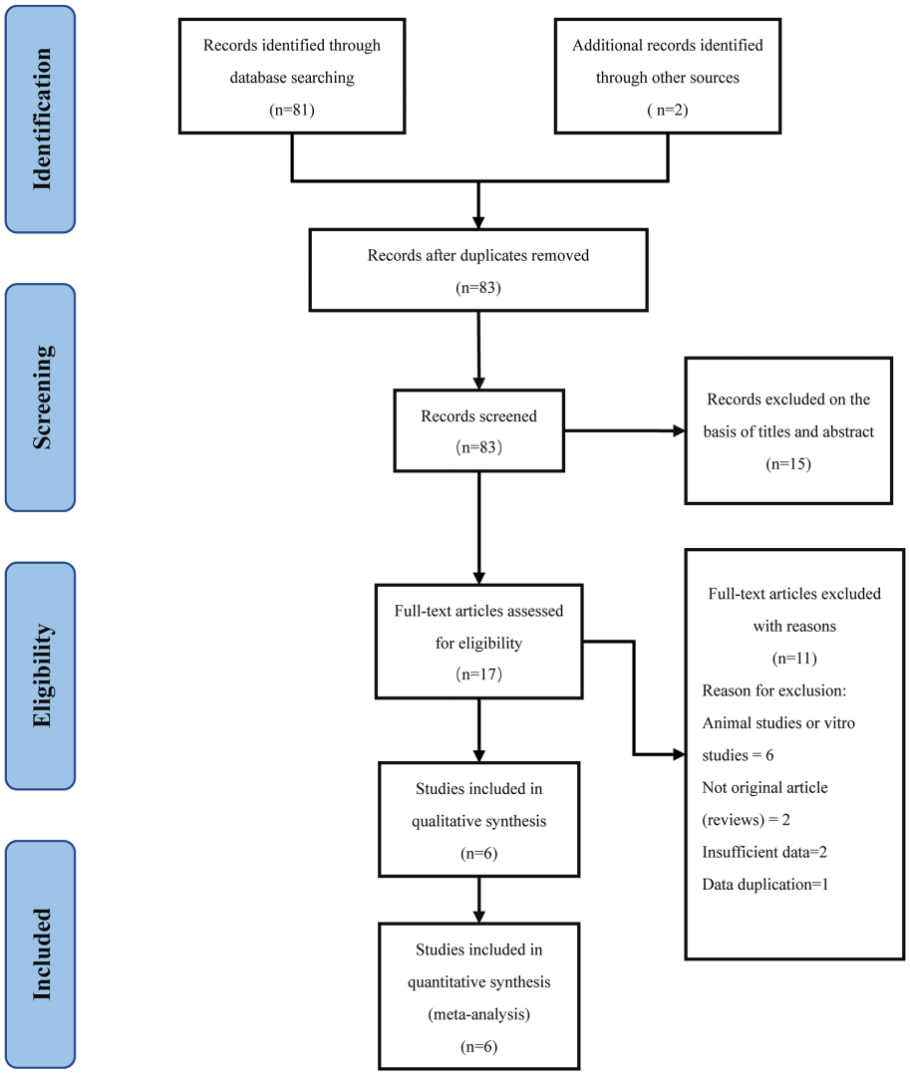
PRISMA diagram.

The exclusion criteria were as follows: 1. The studies are animal studies or vitro studies. 2. Studies without a suitable comparison group. 3. Studies with incomplete or inadequate data. 4. Study type was a review, comment, abstract, case report or irrelevant records. 5. Duplicated publications. 6. Outcome of interest not described (Figure 1).

### Data extraction

To ensure the accuracy and reliability of the data, the two researchers (LY and PJ) collated and evaluated the data independently. The data were extracted from the original manuscript by using a standardized reporting forms designed in advance, including the authors’ name, publication date, fund, baseline data (average age, gender ratio, etc.,), inclusion and exclusion criteria, treatment, total number of cases group and control group, total sample size, number of incident cases, pneumonia and urinary tract infection rate, and sepsis or other infection rates. In addition, both authors assessed the quality item and disagreements or discrepancies were resolved by consensus involving a third author (XW).

### Study endpoints

We considered SAI as the primary outcome, and three common post-stroke infections of stroke associated pneumonia, urinary tract infection, and sepsis as secondary outcomes. We respectively analyzed the protective effects of beta blockers treatment on primary and secondary outcomes.

### Quality assessment

Two authors (WJ and LY) conducted an independent assessment of the risk of bias using the Newcastle-Ottawa Scale (NOS) to assess the quality of the included studies. Sensitivity analysis was performed by excluding studies one by one. Publication bias was evaluated using the Egger test, and *P* < 0.05 was considered significant.

### Data synthesis

RevMan5.3 and Stata 15.0 were used for statistical analysis. The included literature was assessed for quality, heterogeneity test and analyzed for publication bias. Enumeration data were analyzed according to the odds ratio (OR) and 95% CI. If I^2^ < 50%, the heterogeneity among the research results is deemed to be acceptable, the fixed effect model was selected. If I^2^ >50% was considered a high heterogeneity. the random effect model was selected, and the source of heterogeneity was analyzed. In addition, the factors that may lead to heterogeneity were analyzed by subgroup analysis.

## RESULTS

### Article selection

A total of 83 potentially relevant publications were identified in the initial search, of which 2 articles were searched from the reference list [22, 23], and 66 unrelated or unqualified publications were removed by reading the title and abstract. Eleven articles were excluded after full-text reading. Finally, 6 trials were enrolled in pool analysis (Figure 1).

### Article characteristics

The information of characteristics articles is illustrated in Table 1. The rate of SAP, UTI and sepsis for each article is reported in Table 2. A total of 9870 participants were included in this study (a total of 1878 cases and 7992 controls), and the sample size ranged from 138 to 5212 subjects. The mean age ranged between 55–77 years old. The percentage of males ranged from 36.7%–73.4%. In the beta blockers treatment group, most patients had the diseases of the circulatory system, such as hypertension, coronary heart disease and atrial fibrillation (Tables 1 and 2). Four studies included patients with ischemic stroke [9, 16, 18, 24], one study included patients with hemorrhagic stroke [25], and the other had no restrictions on stroke types [17]. All six included studies described pneumonia outcomes [9, 16–18, 24, 25]; three studies described UTI outcomes [9, 17, 24]; and two studies involved bacteremia or septicemia outcomes [9, 24]. In addition, three studies did not avoid the treatment of antibiotics [9, 17, 25], and the other included studies did not mention whether patients were restricted the treatment of antibiotics (Table 2) [16, 18, 24].

**Table 1:** Characteristics of included study.

**Author (Year of publication)**	**Country**	**Inclusion Criteria**	**Study Design**	**Mean Age BB/NB**	**Male BB/NB (%)**	**Ischemic heart diseases BB/NB**	**Myocardial Infarction BB/NB**	**Hypertension BB/NB (%)**	**Atrial Fibrillation BB/NB**	**NIHSS BB/NB**	**NOS Scores**
Ilko L. Maier (2018)	Germany	Ischemic Stroke	Case-control Study	77/69	36.7/59.5	N	36.5/11.6	N	57.9/27.4	17/15	8
Jayantee Kalita (2013)	India	Hemorrhagic Stroke	Case-control Study	55.6/59.7	62.7/73.4	N	12.7/23.7	N	N	N	6
Starr, J. B (2017)	America	Ischemic Stroke	Case-control Study	66.3/61.1	60.4/59.7	21.6/16.9	N	68.1/62.5	11.7/19.6	8/5	7
Sykora, M (2015)	Germany	Ischemic Stroke	Case-control Study	69/68	N	41/29.3	12.9/19.3	96.3/73.2	25.4/25.6	12/11	5
Tomasz Dziedzic (2007)	Poland	Ischemic Stroke	Case-control Study	67.2/69.2	44.3/47	69.3/57.3	25/11.7	81.8/66.3	28.4/20	N	6
Willeke F. Westendorp (2016)	The Netherlands	Ischemic or Hemorrhagic Stroke	Cohort study	77/71	53.8/58.6	N	7/23	79/42	N	5/5	8

Abbreviations: Country: The country of the research institution; BB: beta-blockers; NB: No-beta-blockers; NIHSS: National Institute of Health stroke scale; N: Not given; NOS: Newcastle-Ottawa Scale.

**Table 2:** Infections rate and antibiotic therapy.

**Author (Year of publication)**	**Case number**	**SAP BB/NB (%)**	**UTI BB/NB (%)**	**Sepsis BB/NB (%)**	**Treatment with antibiotic**
Ilko L. Maier (2018)	306	73.41/66.67	31.64/26.14	13.9/1.31	N
Jayantee Kalita (2013)	138	8.86/30.50	N	N	Yes
Starr, J. B (2017)	848	14.86/9.67	9.91/6.13	1.32/0	Yes
Sykora, M (2015)	5212	3.69/8.41	N	N	N
Tomasz Dziedzic (2007)	833	4.55/10.86	N	N	N
Willeke F. Westendorp (2016)	2533	18.98/10.86	8.93/5.64	N	Yes

Abbreviations: SAP: stroke associated pneumonia; UTI: urinary tract infection; BB: beta-blockers; NB: No-beta-blockers; N: Not given.

### Assessment of risk of bias

Six included articles underwent quality assessment according to the Newcastle-Ottawa Scale (Supplementary Tables 3 and 4). This scale consists of 3 parts: the quality of selection, comparability, and outcome. All included studies received more than 5 starts, meaning they had a medium to high quality level (Supplementary Tables 3 and 4).

High heterogeneity was found in some of the pooled analysis. Significant heterogeneity encountered perhaps due to the inability to control for potential confounding factors, such as differences between the various regimens, doses, and duration of beta blockers, as well as different inclusion populations.

Publication bias was evaluated by the Egger method (*P* = 0.585), indicating that there was no significant publication bias. Thus, publication bias had limited effect on the evaluation of results. In addition, sensitive analysis was conducted by removing one study at a time, we recalculated the combined estimate on remaining studies, the combined OR of overall risk estimate were consistent and without apparent fluctuation.

### Primary outcome measure: all infection

**Figure 2:**
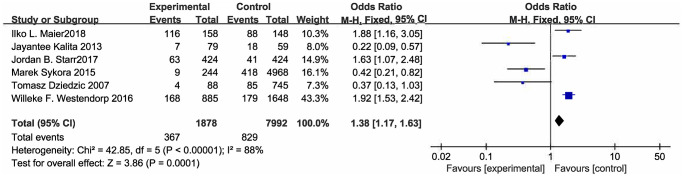
Forest plot of stroke associated infection.

The meta-analysis did not show a significant protective effect of beta blockers on SAI (OR [95% CI] = 0.87 [0.47–1.60], *P* = 0.65) (Figure 2; Table 3). The studies proved to be heterogeneous. Sensitivity analysis and subgroup analysis were performed to explore the sources of heterogeneity. The sensitivity analysis confirmed the stability of the results, indicating that the combined OR was stable. Subgroup analysis was conducted according to study type, stroke type and stroke severity (Table 3). In the pooled analysis, no significant differences were found between the experimental group and the control group.

**Table 3:** Summary of the meta-analysis results.

**Analysis**	**NU**	**Model**	**OR (95% CI)**	**I^2^ (%)**	***P***
Stroke associated infections	6	Rem	0.87 [0.47, 1.60]	88	0.65
Subgroup1: Stroke type					
Ischemic stroke	4	Rem	0.53 [0.18, 1.55]	88	0.24
Hemorrhage stroke	1	Fem	0.22 [0.09, 0.57]	-	0.002
Both type	1	Fem	1.92 [1.53, 2.42]	-	<0.00001
Subgroup2: Study design					
Case-control study	5	Rem	0.71 [0.33, 1.53]	90	0.26
Cohort study	1	Fem	1.88 [1.16, 3.05]	-	<0.00001
Subgroup3: NIHSS					
NIHSS>10	2	Rem	0.90 [0.20, 4.05]	92	0.89
5<NIHSS<10	2	Fem	1.85 [1.51, 2.26]	0	<0.00001
N	2	Fem	0.28 [0.14, 0.56]	0	0.00008
Stroke associated pneumonia	6	Rem	0.76 [0.38, 1.51]	86	0.43
Subgroup1: Stroke type					
Ischemic stroke	4	Rem	0.77 [0.38, 1.54]	84	0.46
Hemorrhage stroke	1	Fem	0.22 [0.09, 0.57]	-	0.002
Both type	1	Fem	1.92 [1.39, 2.65]	-	<0.0001
Subgroup2: Study design					
Case-control study	5	Rem	0.61 [0.31, 1.20]	79	0.15
Cohort study	1	Fem	1.92 [1.39, 2.65]	-	<0.0001
Subgroup3: NIHSS					
NIHSS>10	2	Rem	0.69 [0.47, 1.02]	79	0.06
5<NIHSS<10	2	Fem	1.85 [1.51, 2.26]	0	<0.0001
N	2	Fem	0.29 [0.14, 0.60]	0	0.0008
UTI	3	Fem	1.69 [1.33, 2.14]	0	<0.0001
Sepsis	2	Fem	1.35 [0.71, 2.54]	27	0.36

Abbreviations: NU: number of articles; Rem: random effects models; Fem: fix effects models; OR: odd ratios; CI: confidence interval; Both type: patients with ischemic or hemorrhage stroke; N: not given.

### Secondary outcome measures

**Figure 3:**
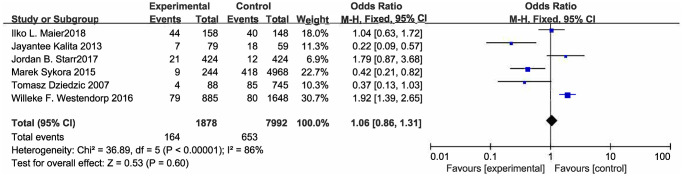
Forest plot of pneumonia.

No evidence could indicate that beta blockers could prevent SAP (OR [95% CI] = 0.76 [0.38. −1.51], *P* = 0.43) (Figure 3; Table 3), but there was high heterogeneity in the results. Heterogeneous sources were explored with subgroup analysis (Shown in Table 3). We found that in patients with stroke with an NIHSS score of 5 to 10, there may have a relationship between the uptake of beta blocker and the occurrence of SAP (OR [95% CI] = 1.85 [1.51, 2.26], *P* < 0.0001).

**Figure 4:**
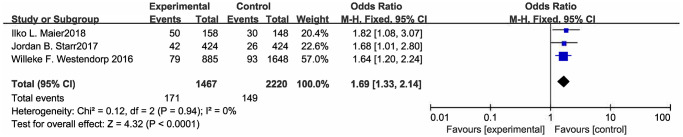
Forest plot of UTI.

As illustrating in Figure 3, it is worth noting that beta blocker treatment is associated with urinary tract infections after stroke (OR [95% CI] = 1.69 [1.33, 2.14], *P* < 0.0001) (Figure 4; Table 3). With regards to the post stroke septicemia or bacteremia, beta blockers also had no obvious protective effect (Supplementary Figure 1; Table 3) (OR [95% CI] = 1.08 [0.60, 1.96], *P* = 0.26).

## DISCUSSION

By performing a meta-analysis of including studies, we found that no significant association between beta blockers therapy and the prevention of post-stroke infection. However, beta blockers may be associated with an increased risk of urinary tract infections and pneumonia in some patients after stroke. However, the current meta-analysis is mainly based on the case-control study by previous publications. Thus, our study calls for caution when assessing the result.

### Stroke-associated infection

We found that simply using beta blockers to prevent SAI did not attain satisfactory results. This result may be attributed to the fact that SAI is determined by various causes or factors, and SIDS is just one of them. Moreover, hyperactivity of SNS does induce SIDS, but reduce SNS activation by beta blockers may not be far enough. Notably, uptake of beta blockers was mostly decided by cardiovascular disease of patients prior to stroke onset. As atrial fibrillation, hypertension and coronary heart disease is closely related to the incidence of stroke, patients with these comorbidities generally have a higher risk of stroke and a dismal prognosis. Maja Wästfelt [26] report that cardiac diseases such as atrial fibrillation may increase the risk of stroke associate infections, therefore, because of the differences between comorbidities, it is possible that the protective effects of beta blockers are obscured. Besides, catecholamines modulate immune cells primarily through the β2-AR [9], but the included studies did not limit the definitive types of receptor blockers, this provides another possible explanation [9]. Besides, three of the included studies did not limit the treatment of antibiotics [9, 17, 25]. It is not yet clear that the mechanisms of the synergistic combination of beta blockers and antibiotics. However, two studies, which are relevant to the efficacy of prophylactic antibiotics on post-stroke infections, found that preventive antibiotics treatment was not recommended for the stroke patients because it may not decrease the risk of infection [27, 28]. Therefore, the treatment of antibiotics may have little effect on the study results, but more studies are necessary in further studies.

In subgroup analysis, it was found that in different stroke types, the use of beta blockers in patients with ischemic stroke may prevent the SAI, while in other type of stroke have no significant prevention effect. But due to the small sample size of other types of strokes, further analysis could not be performed. In subgroup analyses, we found that beta blockers in ischemic stroke may have benefited patients, but further analysis was not possible due to the small sample size of other types of strokes.

### Urinary tract infection

UTIs affect between 10% and 19% of overall stroke patients [29]. It has been found that impaired sympathetic nerve function may cause damage to bladder function and eventually induce urinary tract infection [18, 30]. However, we found that beta blocker treatment is associated with the high post stroke UTI incidence. This contrary conclusion to expectations may be due to the following reasons: first, due to lack of unified diagnostic criteria. Some studies probably include asymptomatic bacteriuria patients and may have affected the results [9]. Second, some of the included patients were treated with antibiotics [27, 28]. Although some research findings antibiotics could reduce rather than increase the occurrence rate of UTI, there are no studies of combination use of beta blockers and antibiotics. Besides, Stott et al.'s prospective study found that catheterization is an independent risk factor for UTI [29, 31]. Thus, indwelling urinary catheters after stroke may be associated with urinary tract infection. This may be one of the confounding factors, Because the data of indwelling urinary catheters were not provided in the included literature, further analysis could not be performed.

### Stroke-associated pneumonia

Our study did not conclude an association between beta blockers and prevention of stroke associated pneumonia. But the results of the pooled analysis showed a significant heterogeneity. Subgroup analysis reduces some heterogeneity. In the subgroup of NIHSS 5-10, beta blockers treatment is associated to the higher risk of stroke associated pneumonia, but this result should be treated with caution due to the absence of data on gastric indwelling, swallowing assessment, etc. This is because current studies have found that risk factors for SAP include swallowing dysfunction, proton pump inhibitor treatment, nasogastric tube placement, and mobility disorders. Notably, aspiration due to swallowing dysfunction is most closely associated with SAP [32–34]. In the subgroup of NIHSS >10, beta blockers had no significant protective effect, this could be attributed to the fact that patients with NIHSS>10 are generally less conscious and have poorer swallowing function, so there is a greater risk of aspiration. Therefore, early assessment and screening for dysphagia are important approaches for the prevention of SAP, and treatment with beta blockers to of inhibiting sympathetic excitation has limited effect.

### Bacteremia or septicemia

This meta-analysis could not corroborate the protective effect of beta blockers treatment on bacteremia or septicemia after stroke. One possible explanation is because septicemia or bacteremia is a state of infection spreading, indicating that the infection is severe [35]. Thus, it may not be possible to control the progression of the infection through limited neuroimmunomodulation.

### Limitations

This study was limited in several ways. First, the number of included studies is relatively small, and retrospective studies account for the majority of included studies. Thus, the quality of the supporting evidence is lower than expected. Second, the potential confounding factors such as the varying dose and treatment period of beta blockers and the combination with antibiotics also increase the heterogeneity among the included study. Most of the included articles were from Western countries. Therefore, there may be bias in the article due to racial/ethnic differences, so the results cannot be directly extrapolated to the eastern population. Although we attempted to reduce racial difference by searching database in other languages, due to the language limitations, only databases in English and Chinese were concluded in this study, which means there may have studies that was not assessable for pool analysis. Third, the included studies suffer from significant sources of bias, which means there is substantial heterogeneity among studies. Besides, subjects in the comparison group received antibiotics therapy or other antihypertensive medication which can also influence outcome. Due to the existence of the above limitations, the evidence to support it is low, and the conclusion should be treated with caution.

## CONCLUSIONS

This meta-analysis cannot draw robust conclusions on the protective effect of beta blocker therapy on stroke associated infection. Besides, the result suggests that beta blockers treatment may be associated with development of post-stroke urinary tract infection. Besides, in the patients with mild to moderate stroke, the uptake of beta blocker may have an adverse effect on the prevention of stroke associated pneumonia.

## Supplementary Materials

Supplementary Figure 1

Supplementary Table 1

Supplementary Table 2

Supplementary Tables 3 and 4
