# Supplementary figures and images for: Are beta blockers effective in preventing stroke-associated infections? - a systematic review and meta-analysis
Source: Aging (Albany NY). 2022 May 18;14(10):4459–70. doi: 10.18632/aging.204086 (PMC9186777; doi:10.18632/aging.204086)

SUPPLEMENTARY FIGURE

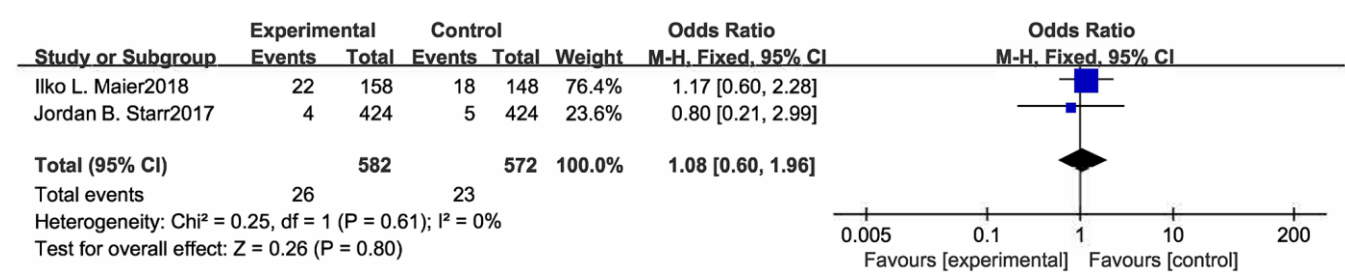

Supplementary Figure 1. Forest plot of bacteremia or septicemia.

Supplement: Supplementary Figure 1 [file aging-14-204086-s001.pdf]
