# Supplementary material for: Are beta blockers effective in preventing stroke-associated infections? - a systematic review and meta-analysis
Source: Aging (Albany NY). 2022 May 18;14(10):4459–70. doi: 10.18632/aging.204086 (PMC9186777; doi:10.18632/aging.204086)
Supplement: Supplementary Table 2 [file aging-14-204086-s003.docx]

**Supplementary Table 2. Search Strategy in PubMed.**

Up to 2022/2/10

|  | Strategy | Number |
| --- | --- | --- |
| #1 | "Stroke"[Mesh] OR "Hemorrhagic Stroke"[Mesh] OR "Thrombotic Stroke"[Mesh] OR "Ischemic Stroke"[Mesh] OR "Brain Stem Infarctions"[Mesh] OR "Infarction, Posterior Cerebral Artery"[Mesh] OR "Infarction, Middle Cerebral Artery"[Mesh] OR "Infarction, Anterior Cerebral Artery"[Mesh] OR "Embolic Stroke"[Mesh] OR"Intracranial Thrombosis"[Mesh:NoExp] | 155636 |
| #2 | "Brain Ischemia"[Mesh] OR "Cerebral Hemorrhage"[Mesh] OR "Basal Ganglia Cerebrovascular Disease"[Mesh] | 147767 |
| #3 | Stroke*[Title/Abstract] OR Cerebrovascular Accident*[Title/Abstract] OR "Cerebrovascular Apoplexy"[Title/Abstract] OR Brain Vascular Accident*[Title/Abstract] OR Hemorrhagic Stroke*[Title/Abstract] OR Thrombotic Stroke*[Title/Abstract] OR Ischemic Stroke*[Title/Abstract] OR Brain Infarction*[Title/Abstract] OR Brain Hemorrhage*[Title/Abstract] OR Brain Infarction*[Title/Abstract] | 292321 |
| #4 | #1 OR #2 OR #3 | 394710 |
| #5 | "Pneumonia"[Mesh] OR "Pneumonia, Ventilator-Associated"[Mesh] OR "Pneumonia, Staphylococcal"[Mesh] OR "Pneumonia, Bacterial"[Mesh] OR "Pneumonia, Mycoplasma"[Mesh] OR "Pneumonia, Pneumococcal"[Mesh] OR "Pneumonia, Aspiration"[Mesh] OR "Healthcare-Associated Pneumonia"[Mesh] OR "Bronchopneumonia"[Mesh] OR "Urinary Tract Infections"[Mesh] OR "Sepsis"[Mesh] OR "Sepsis-Associated Encephalopathy"[Mesh] OR "Systemic Inflammatory Response Syndrome"[Mesh] OR "Bacteremia"[Mesh] OR "Fungemia"[Mesh] | 404442 |
| #6 | Infection*[Title/Abstract] OR Infestation*[Title/Abstract] OR Lung Inflammation*[Title/Abstract] OR Bronchopneumonia*[Title/Abstract] OR Bacteremia*[Title/Abstract] OR Bloodstream Infection*[Title/Abstract] OR Septicemia*[Title/Abstract] OR Blood Poisoning*[Title/Abstract] OR Endotoxin Shock*[Title/Abstract] OR Urinary Tract Infection*[Title/Abstract] | 1630251 |
| #7 | #5 OR #6 | 1875398 |
| #8 | #4 AND #7 | 13534 |
| #9 | "Stroke associated pneumonia"[Title/Abstract] OR stroke associated infection*[Title/Abstract] | 210 |
| #10 | #8 OR #9 | 13577 |
| #11 | "Adrenergic beta-Antagonists"[Mesh] OR "Adrenergic beta-2 Receptor Antagonists"[Mesh] OR "Adrenergic beta-1 Receptor Antagonists"[Mesh] | 42680 |
| #12 | β-blocker*[Title/Abstract] OR Carvedilol[Title/Abstract] OR sotalol[Title/Abstract] OR Atenolol[Title/Abstract] OR Propranolol[Title/Abstract] OR sotalol[Title/Abstract] OR Propranololum[Title/Abstract] OR Metoprolol[Title/Abstract] OR Lopresor[Title/Abstract] OR Betaloc[Title/Abstract] OR Seloken[Title/Abstract] | 74918 |
| #13 | #11 OR #12 | 94962 |
| #14 | #10 AND #13 | 76 |
|  |  |  |
