# Supplementary material for: Are beta blockers effective in preventing stroke-associated infections? - a systematic review and meta-analysis
Source: Aging (Albany NY). 2022 May 18;14(10):4459–70. doi: 10.18632/aging.204086 (PMC9186777; doi:10.18632/aging.204086)
Supplement: Supplementary Tables 3 and 4 [file aging-14-204086-s004.pdf]

SUPPLEMENTARY TABLES

Supplementary Table 3. Result of quality assessment using the Newcastle-Ottawa scale for cohort study.

Author (Year)	Selection				Comparability of cohorts on the basis of the design or analysis	Exposure			Scores
	Representat-iveness of the exposed cohort	Selection of the non-exposed cohort	Ascertainment of exposure	Demonstration that outcome of interest was not present at start of study		Assessment of outcome	Was follow-up long enough for outcome to occur	Adequacy of follow up of cohort	
Westendorp, W. F (2016)	☆	☆	☆	☆	☆☆	☆	☆	-	8

Supplementary Table 4. Result of quality assessment using the Newcastle-Ottawa scale for case-control study.

Author (Year)	Selection				Comparability control for important factor	Exposure			Scores
	Adequate definition of cases	Represe-ntativeness of the case	Selection of controls	Definition of controls		Ascertainmen t of exposure	Same method of ascertainment for cases and controls	Non-response rate	
Maier, I. L (2018)	☆	☆	☆	☆	☆☆	☆	☆	–	8
Kalita, J (2013)	☆	☆	☆	–	☆	☆	☆	–	7
Starr, J. B (2017)	☆	☆	☆	☆	☆	☆	☆	–	6
Sykora, M (2015)	–	☆	–	☆	☆	☆	☆	–	5
Dziedzic, T (2007)	☆	☆	☆	☆	☆	-	☆	–	6
